# Recovery from COVID-19: a 12-month follow-up study on cardiorespiratory fitness and pulmonary function

**DOI:** 10.3389/fcvm.2025.1638317

**Published:** 2025-08-12

**Authors:** Cássia da Luz Goulart, Vinícius Maldaner, Carla Cristina de Araújo Alves, Mauricio Milani, Juliana Milani, Ana Clara Gonçalves da Costa, Marcela Lopes Alves, Robson Fernando Borges, Claudia Cristina Conde Holanda Sobral, Letícia de Araújo Moraes, Jean Carlos Coutinho, Nádia Oliveira Gomes, Leandro Tolfo Franzoni, Filipe Ferrari, Audrey Borghi-Silva, Lawrence P. Cahalin, Graziella França B. Cipriano, Gerson Cipriano Junior, Ricardo Stein

**Affiliations:** ^1^ Health Sciences and Technologies Post Graduate Program, University of Brasilia (UnB), Brasilia, Brazil; ^2^ Cardiopulmonary Physiotherapy Laboratory, Physiotherapy Department, Federal University of Sao Carlos, UFSCar, Sao Carlos, Brazil; ^3^ Rehabilitation Sciences Post Graduate Program, University of Brasilia (UnB), Brasilia, Brazil; ^4^ Graduate Program in Human Movement and Rehabilitation, Evangelical University of Goias, Hasselt, Brazil; ^5^ Rehabilitation Research Center (REVAL), Faculty of Rehabilitation Sciences, Hasselt University, Hasselt, Belgium; ^6^ Department of Cardiology, Heart Centre Hasselt, Jessa Hospital, Hasselt, Belgium; ^7^ Department of Clinical Medicine, Federal University of Rio Grande do Sul, Porto Alegre, Brazil; ^8^ Department of Physical Therapy, Miller School of Medicine, University of Miami, Miami, FL, United States; ^9^ Postgraduate Program in Health Science, Cardiology and Cardiovascular Sciences, Hospital de Clinicas, Porto Alegre, Brazil

**Keywords:** COVID-19, CPET, lung function, functional capacity, recovery

## Abstract

**Introduction and aim:**

Long COVID, characterized by persistent symptoms after acute infection, poses a major public health challenge. Understanding its long-term effects is crucial, particularly in relation to cardiorespiratory recovery. This study aimed to assess changes in cardiorespiratory fitness (CRF) and pulmonary function (PF) over 12 months following acute COVID-19, addressing a significant gap in current knowledge about the disease's lasting impact.

**Methods:**

This prospective cohort study included 29 individuals previously diagnosed with post-acute COVID-19. The baseline data were collected during the acute phase of infection*.* Participants underwent clinical evaluation, cardiopulmonary exercise testing (CPET), spirometry, and maximal inspiratory pressure (MIP) measurement at baseline and again after 12 months.

**Results:**

After one-year, significant improvements were observed across several CPET parameters, including VE/MVV ratio (Cohen's *D* = 0.66), peak oxygen uptake (VO_2_peak) in both absolute and relative terms (ml/min: *d* = 0.67; and ml/kg/min: *d* = 0.45), oxygen uptake efficiency slope (OUES; *D* = 0.47) and a reduction in VE/VCO_2_ slope (*D* = 0.80). Pulmonary function improved with increases in % predicted forced expiratory volume in 1 s (FEV_1_; *d* = 0.67) and forced vital capacity (FVC; *D* = 0.67). MIP improved significantly (*D* = 0.67), and the prevalence of inspiratory muscle weakness decreased from 20.7% at baseline to 3.5% at follow-up.

**Conclusion:**

Despite the severity of their initial illness, patients demonstrated substantial recovery in CRF, PF, and inspiratory muscle strength over 12 months.

## Introduction

Post-acute COVID-19 syndrome, or long COVID, is a complex, multisystem condition affecting approximately 39%–46% of individuals who recover from acute SARS-CoV-2 infection (1, 2). Characterized by persistent symptoms such as fatigue, dyspnea, muscle weakness, and exercise intolerance, these manifestations can last for 12 weeks or more, significantly impairing quality of life and placing a substantial burden of healthcare systems (3–6). The heterogeneous nature of long COVID presents significant challenges for healthcare providers, as symptoms can persist or emerge months after the initial infection, regardless of the severity of the acute illness (3, 4).

Among its many effects, long COVID has been associated with reductions in cardiorespiratory fitness (CRF), a well-established indicator of overall health (7–9). Even individuals without prior comorbidities may experience decreased exercise capacity and endurance months after infection, often resulting from combined cardiovascular, pulmonary, and peripheral limitations (3, 4). However, the underlying mechanisms contributing to these persistent functional impairments remain incompletely understood, particularly regarding the specific contributions of respiratory muscle dysfunction and pulmonary function abnormalities.

In addition, respiratory muscle dysfunction – particularly inspiratory muscle weakness – had been identified as a key contributor to reduce exercise tolerance in long COVID. Studies have shown that even patients with mild acute illness may exhibit decreased maximum inspiratory pressure (MIP) and phrenic nerve dysfunction, despite the absence of overt (5, 6). The prevalence of pulmonary function abnormalities varies considerably across studies, with reports indicating that 26% of long COVID patients present evidence of reduced pulmonary function. A significant proportion of COVID-19 survivors continue to experience reduced diffusion capacity three and twelve months after discharge, highlighting the persistent nature of these impairments (10). These findings underscore the critical need for comprehensive pulmonary function assessment in long COVID patients, as traditional spirometric measures may not capture the full extent of respiratory impairment (11).

Given the high prevalence and multifactorial nature of long COVID (3, 12), this study, therefore, aimed to evaluate changes in cardiorespiratory fitness, pulmonary function, and respiratory muscle strength over a 12-month period following acute COVID-19 infection.

## Methods

### Study design and settings

This prospective cohort study was conducted in accordance with the STROBE statement (13) and involved a 12-month follow-up period of individuals previously infected with COVID-19. The patients were evaluated at baseline (during the acute phase of infection in 2020) and 12 months later post the acute phase of infection. Data were collected between 2020 and 2021 at the Laboratory of Clinical Exercise Physiology, University of Brasilia.

Eligible patients were referred for outpatient evaluation and underwent standardized assessment as part of their clinical follow-up. The study protocol was approved by the Medical Ethics Committee of the University of Brasilia (CAAE: 35706720.4.0000.8093), and all procedures adhered to national and international ethical guidelines for human research.

### Participants

Adults aged ≥18 years with a confirmed history of symptomatic COVID-19 – diagnosed via reverse transcription-polymerase chain reaction (RT-PCR) – were eligible for inclusion. Participants with pre-existing cardiovascular or pulmonary diseases, clinical instability or conditions that could compromise functional test performance were excluded.

All participants underwent a comprehensive medical evaluation, and COVID-19-specific clinical data were collected through standardized assessments. The severity of the acute illness was classified according to the World Health Organization (WHO) provisional clinical guidance (14).

### Recruitment strategy and participant inclusion

Participants were recruited through targeted outreach on social media platforms and via contacts from related studies. Eligible individuals were invited to undergo a series of standardized clinical assessments, including cardiopulmonary exercise testing (CPET), spirometry, and evaluation of inspiratory muscle strength.

### Clinical assessments

Collected data included detailed history of COVID-19 infection, hospitalization and discharge information, severity of the acute illness, medical history, and existing comorbidities. These evaluations provided the basis for participant characterization and stratification in subsequent analyses.

### Cardiopulmonary exercise testing

After a familiarization session, participants underwent CPET during their first visit laboratory using an electronically braked cycle ergometer (Corival 400, Lode, The Netherlands). A ramp-incremental exercise protocol (5–15 W/min) was employed at a constant cadence of 60 rpm, following a 2-minute warm-up period at 0 W.

Oxygen uptake (VO_2_) and carbon dioxide production (VCO_2_) in ml/min, minute ventilation (VE, L/min), and the oxygen uptake efficiency slope (OUES) were measured using a breath-by-breath gas analysis system (Quark CPET, Cosmed, Italy), accompanied by continuous 12-lead electrocardiogram monitoring for heart rate (HR, bpm) (15).

The ventilatory threshold was identified visually using the V-slope method by examining the inflection point on the VCO_2_ vs. VO_2_ plot. Maximal effort was confirmed based on the highest 30 sec averaged VO_2_ and a peak respiratory exchange ratio (RER) ≥ 1.10 (16).

Predicted values for VO_2_peak (17) and OUES (18) were calculated using reference equations specific to the Brazilian population. Indirect maximal voluntary ventilation (MVV) was estimated as FEV_1_ × 40. Breathing reserve (%) was calculated using the following formula: breathing Reserve (%) = [(MVV − VE/MVV) × 100 (8).

### Lung function by spirometry

Spirometry was performed using a MicroQuark spirometer (Cosmed, Milan, Italy) in accordance with the American Thoracic Society (ATS) guidelines (19). The following variables were assessed: forced expiratory volume at the first second (FEV_1_), forced vital capacity (FVC), and the FEV_1_/FVC ratio. Predicted values were based on reference equations for the Brazilian population (20). To ensure safety and prevent cross-contamination between participants and equipment, specific disposable antibacterial/antiviral filters were used during testing.

### Respiratory muscle strength by manovacuometry

Inspiratory muscle strength was assessed using a digital manovacuometer (MVD300-U, Globalmed®, Brazil) to measure MIP, following the recommendations of the ATS/European Respiratory Society (21). Predicted MIP values were derived from Sclauser Pessoa et al. (22), and the criteria for identifying inspiratory muscle weakness (IMW) were defined according to Rodrigues et al. (23).

## Statistical analysis

Data are presented as mean ± standard deviation (SD), median with interquartile range (IQR), percentages, and 95% confidence intervals (Cis), as appropriate. Normality of the data was assessed using the Shapiro–Wilk test. For within-group comparisons, paired t-tests or Wilcoxon signed-rank tests were applied, depending on data distribution. Categorical variables were analyzed using the McNemar Test. Effects size were calculated using Cohen's D and interpreted as small (0.2–0.49), medium (0.5–0.79), and large (≥0.8) (24). All analyses were conducted using SigmaPlot (Systat Software, Chicago, USA), IBM SPSS Statistics for Mac (version 24.0), and GraphPad Prism (version 7.0, USA).

## Results

**Figure 1:**
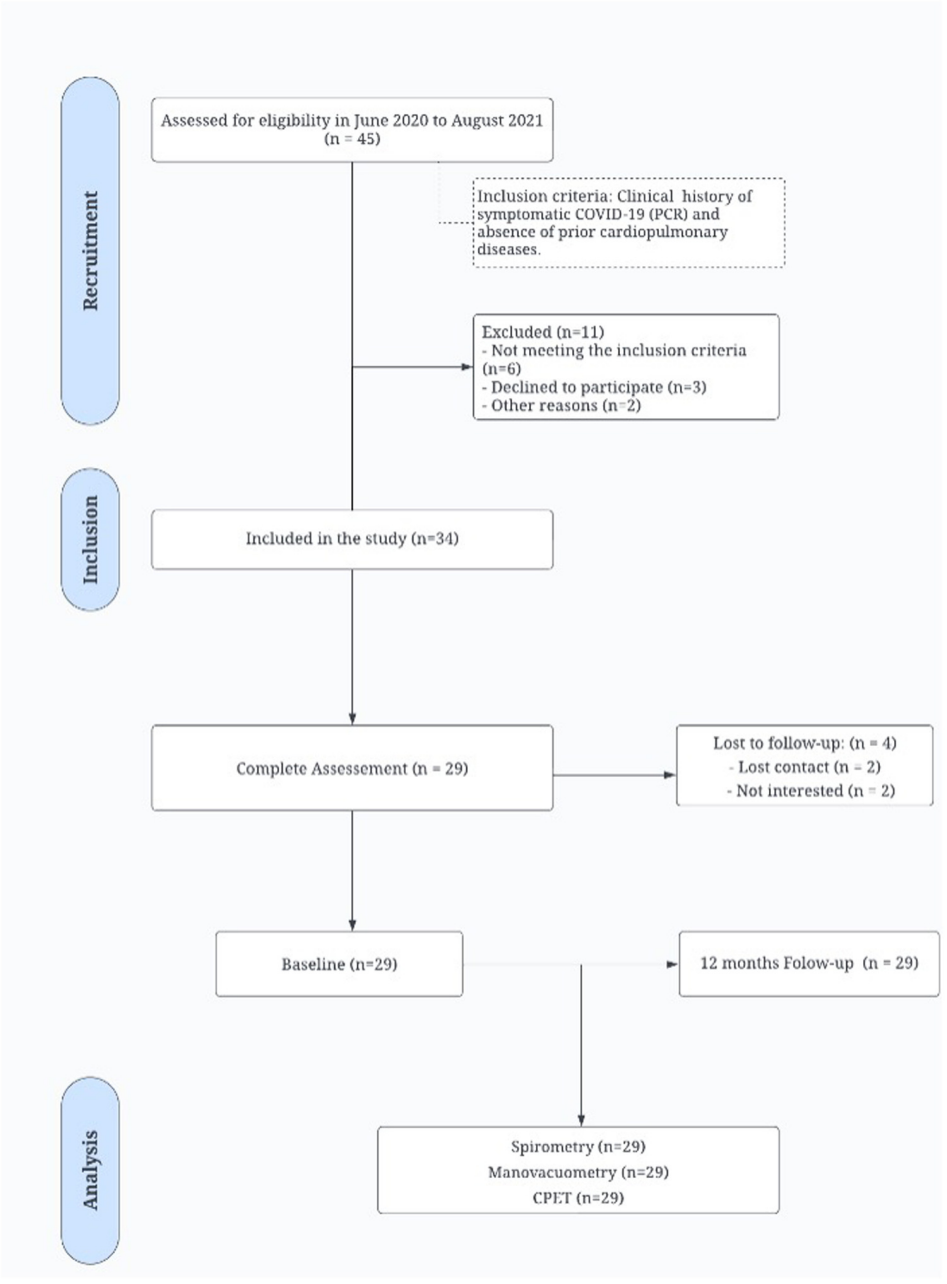
STROBE flow chart. This chart illustrates the patient identification, inclusion, follow-up, and analysis process in the study. CPET, Cardiopulmonary exercise testing.

We initially recruited 45 patients diagnosed with acute COVID-19. However, due to stringent inclusion criteria, 11 patients were excluded: 6 did not meet the eligibility criteria, 3 declined participation, and 2 who were lost to follow-up. This resulted in a final cohort of 34 patients, of whom 29 successfully completed all assessments, as illustrated in Figure 1.

A follow-up evaluation conducted 12 months after the initial assessment revealed that most patients had experienced severe illness during the acute phase of COVID-19, with hypertension being the most prevalent comorbidity. Comparisons between baseline and follow-up revealed no significant differences in demographic characteristics (*p* > 0.05), as detailed in Table 1.

**Table 1:** Baseline characteristics of the study participants.

Variables	Baseline (*n* = 29)	Follow-up (*n* = 29)	*P* value
Age (years)	55.0 ± 11.7	56.1 ± 11.5	0.48
Weight (kg)	82.0 ± 17.7	85.0 ± 19.6	0.44
Heigh (cm)	161.9 ± 8.3	161.3 ± 8.4	0.85
BMI (kg/m^2^)	31.6 ± 6.0	32.6 ± 6.4	0.41
Hospitalization time (days)	27.5 ± 19.6	–	–
Smoking	1.0 (3.4)	1.0 (3.4)	0.85
Severity of acute COVID-19, *n* (%)			
Mild	2 (8)	–	
Moderate	1 (4)	–	
Severe	17 (57)	–	
Critical	9 (31)	–	
Comorbidities, *n* (%)			
Hypertension	23.0 (79.3)	20.0 (69)	0.07
Diabetes mellitus	9.0 (31)	8.0 (27.6)	0.92
Dyslipidemia	10.0 (34.5)	12.0 (41.4)	0.90
Cardiac diseases	3.0 (10.3)	–	–

Student's T-test; BMI, body mass index. Data are reported as means ± SD or number and frequency.

As shown in Table 2, CRF analyses at the 12-month follow-up revealed significant improvements in several measures, including an increase in the VE/MVV (Cohen's *D* = 0.66; *p* = 0.001), VO_2_peak in both ml/min and ml/kg/min (*p* = 0.002, Cohen's *D* = 0.67 *p* = 0.037, Cohen's *D* 0.45), and the OUES (*p* = 0.032, Cohen's *D* = 0.47). Additionally, there was a reduction in the VE/VCO_2_ slope (*p* = 0.001, Cohen's *D* = 0.80) (Table 2).

**Table 2:** Pulmonary function, respiratory muscle strength, and cardiopulmonary exercise testing (CPET) at baseline and follow-up.

Variables	Baseline (*n* = 29)	Follow-up (*n* = 29)	Mean difference (CI 95%)	D Cohen	*P* value
CPET					
WR (W)	88 ± 28	113 ± 31	24.5 (17.6–31.4)	1.5	0.04
VE/VCO_2_ slope	35 ± 5	30 ± 4	4.4 (2.13–6.69)	0.8	0.001
VO_2_peak (ml/min)	1.2 ± 0.3	1.6 ± 0.3	0.34 (0.14–0.54)	0.73	0.002
VE/VVM	0.60 ± 0.16	0.81 ± 0.36	0.21 (0.09–0.33)	0.66	0.001
HR peak, beats per minute	138 ± 24	148 ± 20	10.2 (3.5–17.0)	0.64	0.005
OUES	1,679 ± 485	1,923 ± 404	243.9 (22.8–465.0)	0.47	0.032
VO_2_peak (ml/kg/min)	15.6 ± 4.3	19.0 ± 6.6	3.4 (0.22–6.59)	0.45	0.037
VCO_2_peak (L/min)	1.1 ± 0.3	1.2 ± 0.2	0.11 (0.01–0.21)	0.35	0.068
OUES (% of predicted)	57 ± 17	67 ± 26	9.47 (8.1–20.1)	0.31	0.098
VO_2_peak (% of predicted)	28.3 ± 4.0	32.0 ± 2.0	3.6 (3.1–10.2)	0.2	0.287
RER	1.16 ± 0.09	1.18 ± 0.01	0.02 (0.01–0.06)	0.15	0.461
Pulmonary function					
FVC (% predicted)	86 ± 20	99 ± 25	12.3 (4.5–20.1)	0.67	0.003
FVC (L)	2.8 ± 0.7	3.0 ± 0.8	0.26 (0.08–0.45)	0.61	0.006
FEV_1_ (% predicted)	86 ± 14	94 ± 21	8.2 (2.2–14.2)	0.57	0.01
FEV_1_ (L)	2.4 ± 0.1	2.8 ± 0.1	0.76 (0.51–0.21)	0.23	0.254
Respiratory muscle strength					
MIP (cmH_2_O)	78 ± 23	96 ± 27	17.3 (6.4–28.3)	0.67	0.003
MIP (% predicted)	87 ± 9	92 ± 9	20.4 (1.6–39.3)	0.55	0.525

Student's T-test; FEV_1_, forced expiratory volume at the first minute; FVC, forced vital capacity; MIP, maximum inspiratory pressure; HR: VCO_2_, carbon dioxide production; V˙O_2_, oxygen uptake; RER, respiratory exchange ratio; WR, work rate; VE, minute ventilation; VVM, maximal voluntary ventilation; VE/VCO_2_ slope, linear relation between minute ventilation and carbon dioxide production; OUES, oxygen uptake efficiency slope. Data are ordered by the effect size (Cohen's D) from largest to smallest.

Significant improvements were also observed in the % of predicted FEV_1_ and % of predicted FVC (*p* = 0.010, Cohen's *D* = 0.57 and *p* = 0.003, Cohen's *D* = 0.67, respectively) (Table 2).

**Figure 2:**
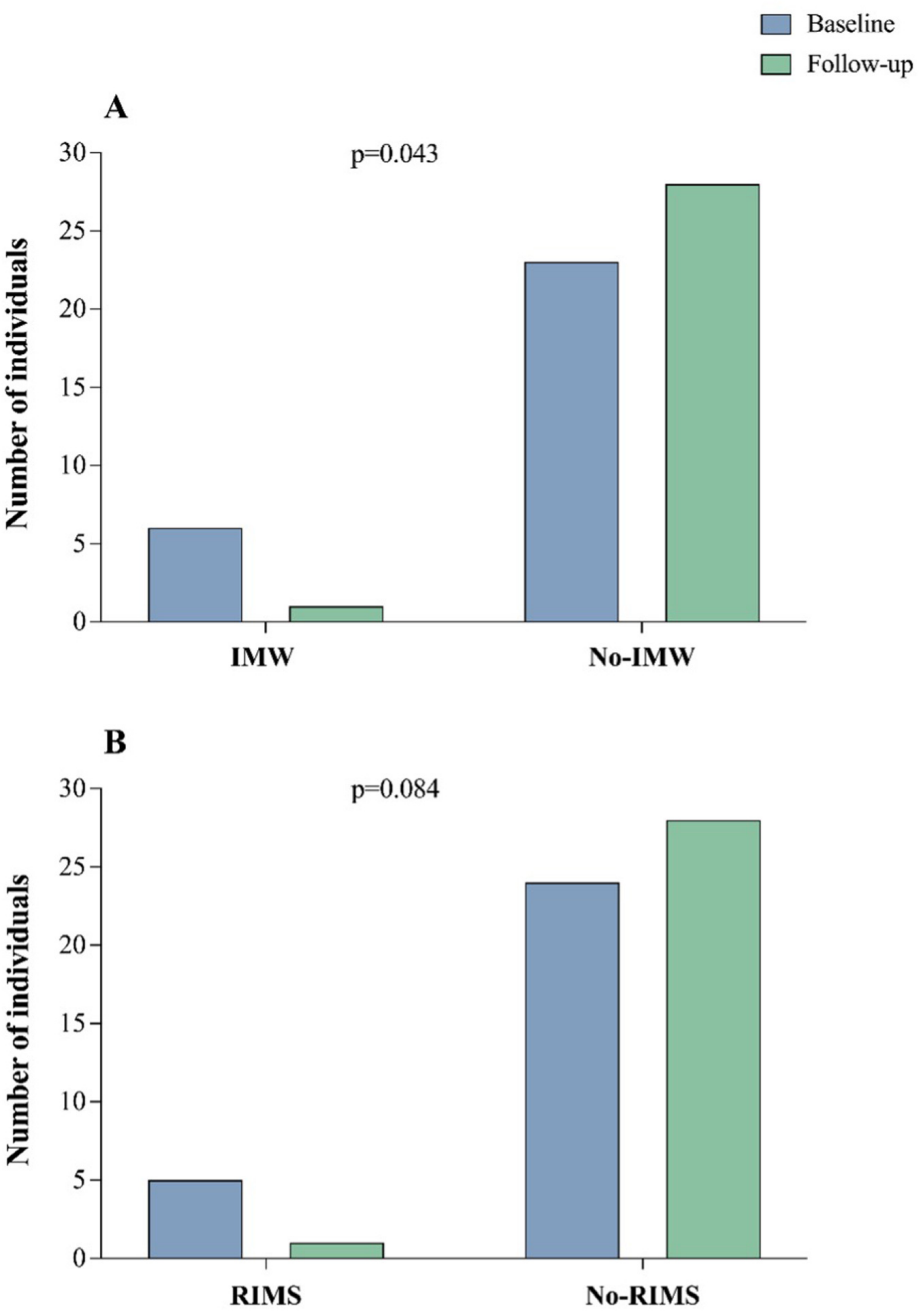
Comparison of inspiratory muscle weakness and reduced inspiratory muscle strength between baseline and follow-up. This figure illustrates in **(A)**: the changes in the prevalence of inspiratory muscle weakness (IMW) and in **(B)**: reduced inspiratory muscle strength (RIMS) from baseline to 12-month follow-up. Statistical analysis was performed using the Chi-square test to evaluate differences between the two-time points.

Table 2 shows that MIP increased significantly from 78 ± 23 to 96 ± 27 cmH_2_O (*p* < 0.05, Cohen's *D* = 0.67). Figure 2 illustrates the prevalence of IMW (22, 23) and reduced inspiratory muscle strength (RIMS) at baseline and 12-months post-COVID. IMW decreased significantly from 20.69% at baseline to 3.45% at follow-up (*p* = 0.007), while RIMS also decreased, approaching significance, from 17.64% at baseline to 3.45% at follow-up (*p* = 0.084).

**Figure 3:**
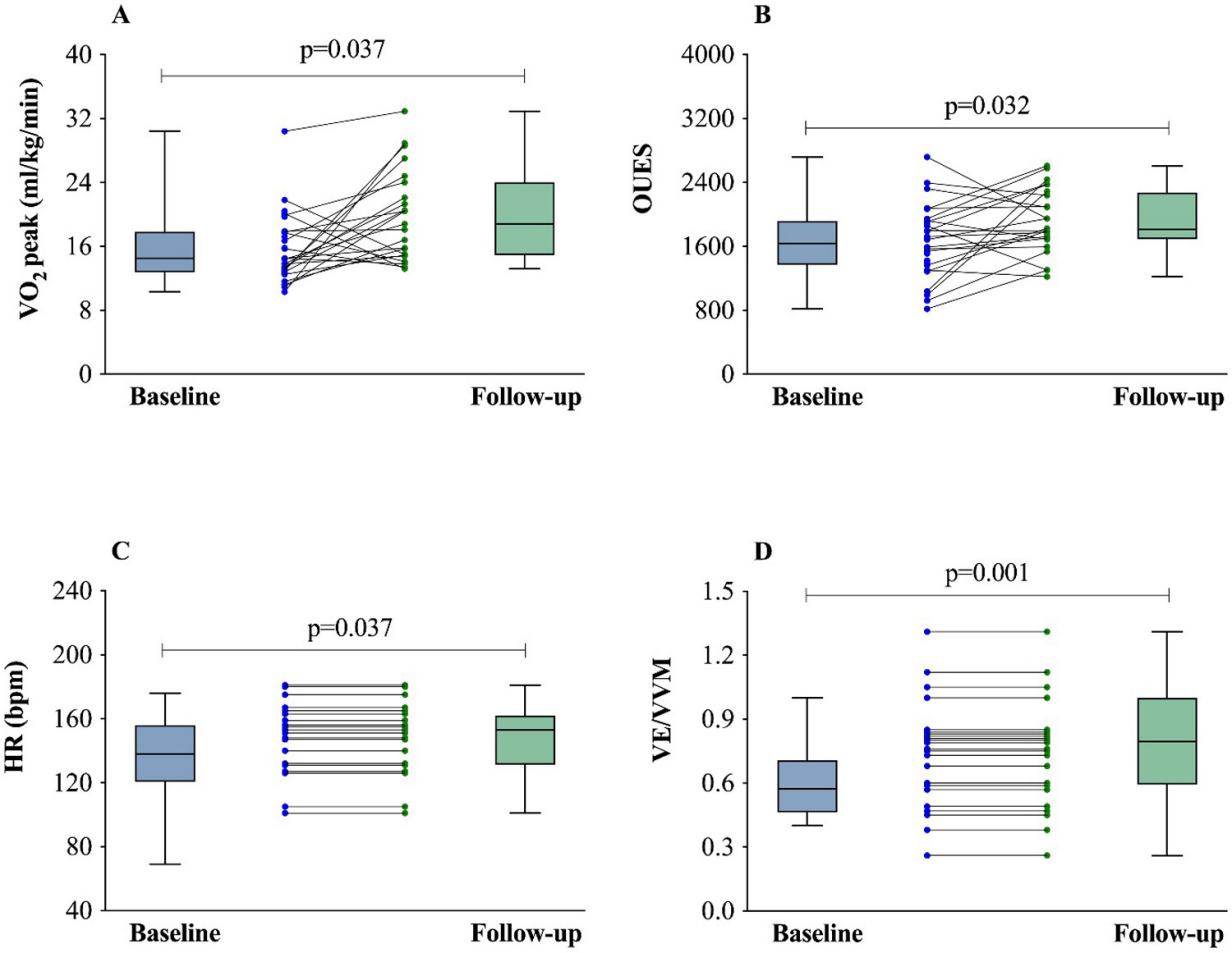
**(A–D)** Comparison between baseline and follow-up of VO_2_ (L/kg/min), OUES, HR peak (bpm), and VE/VVM. OUES, oxygen uptake efficiency slope; VO_2_, oxygen uptake; VE, minute ventilation; VVM, maximal voluntary ventilation; HR: heart rate. Student's T-test.

 Figure 3 provides individual and average responses of key ventilatory, hemodynamic, gas exchange, and oxygen consumption variables after 12 months, highlighting marked improvements in all assessed parameters (*p* < 0.005). These findings underscore the substantial recovery in CRF, pulmonary function, and inspiratory muscle strength in patients following acute COVID-19 infection.

## Discussion

This study thoroughly assessed the long-term effects of COVID-19 survivors, with a specific focus on CRF, pulmonary function, and RIMS over a 12-month period following infection. The results demonstrated significant improvements in these domains, signaling a notable recovery in patients who had experienced acute COVID-19. The main findings of this study were significant improvements in VO_2_ peak, VE/VVM, and OUES, with a reduction in the VE/VCO_2_ slope, which were associated with significant improvements in MIP, FEV_1_, and FVC. Additionally, a decrease in the frequency of IMW from baseline to follow-up was also observed and likely contributed to many of the above findings.

CPET is a comprehensive diagnostic tool that assesses the integration of the cardiovascular, pulmonary, and metabolic systems during physical exercise (25). CPET provides valuable information about functional capacity and the underlying mechanisms of exercise intolerance in different populations, including patients affected by COVID-19 (26). Previous studies have demonstrated changes in pulmonary function, reduced exercise capacity, and abnormalities in the cardiopulmonary response in post-COVID patients, suggesting a lasting and limiting impact of the infection (26).

One of the key findings was the improvement in VO_2_ peak, as it is a fundamental measure of aerobic capacity and a crucial determinant of overall health and functionality (27, 28). The increase in VO_2_ peak, alongside the reduction in the VE/VCO_2_ slope, suggests an enhanced ventilatory efficiency during exercise, possibly reflecting better pulmonary gas exchange and reduced ventilatory demand during exercise (29). Additionally, the improvement in OUES further supports greater cardiovascular and respiratory efficiency, which is likely to result in better outcomes for patients with long COVID (18).

The study also documented significant improvements in FEV_1_ and FVC, indicating greater lung volume and flow in patients who experienced mostly severe COVID-19 and restrictive lung defects commonly observed following severe viral infections. The improvements in these parameters suggest a potential reversal of such defects, which is a positive outcome for patients experiencing prolonged effects from long COVID. Recent studies have demonstrated that post-infection COVID-19 patients frequently show impaired lung function, with the diffusion capacity for carbon monoxide (DLCO) being the most consistently affected parameter (30). While our study did not specifically assess DLCO, the improvements in FEV_1_ and FVC suggest that the restrictive patterns commonly observed in post-COVID patients may be more reversible than initially anticipated.

Improvement in RMS was another critical observation, marked by increased MIP and a reduced frequency of IMW. These enhancements reflect strengthened respiratory muscles, essential for reducing the risk of future respiratory complications and enhancing patients' ability to perform daily activities, there enhancing their quality of life (31). The improvement in ventilatory efficiency, demonstrated by the increase in VE/MVV, indicates better management of ventilatory capacity and reduced hyperinflation (8). This is particularly relevant for long COVID-19 patients, who often face issues with air trapping and lung stiffness.

The observed improvements across various metrics suggest potential benefits from monitoring and supporting natural recovery in COVID-19 survivors. While the data indicate that patients may experience significant enhancements in CRF, pulmonary function, and respiratory muscle strength, caution must be exercised before attributing these outcomes to specific rehabilitation interventions due to the observational nature of this study.

### Strengths and limitations

The limitations of this study include the absence of a control group, which restricts our ability to distinguish the specific impacts attributable solely to COVID-19 from those due to natural recovery processes or other interventions. Additionally, the single-center design may limit the generalizability of the results across different populations and settings. Future research should extend the follow-up period beyond the current 12-month timeframe to deepen our understanding of long COVID. Incorporating additional follow-up assessments at 18, 24 months, and beyond would offer valuable insights into the long-term progression or resolution of symptoms and functional impairments associated with COVID-19. Furthermore, future studies could explore the effectiveness of specific rehabilitation interventions, such as aerobic exercise programs or respiratory muscle training, in improving long-term outcomes in COVID-19 survivors.

## Conclusion

This 12-month observational study highlights both the persistent challenges and the significant potential for recovery in individuals with long COVID. Our findings demonstrate substantial improvements in CRF, pulmonary function, and RIMS. The reduction in inspiratory muscle weakness prevalence from 20.69% to 3.45%, accompanied by significant improvements in MIP, FEV_1_, and FVC, demonstrates that meaningful recovery is possible even in patients who experienced severe acute illness.

These results underscore the possibility of considerable physiological recovery over time, emphasizing the importance of ongoing monitoring and personalized rehabilitation strategies to optimize outcomes in the management of long COVID. The relationship between respiratory muscle strength and overall functional capacity highlights the critical importance of comprehensive respiratory muscle assessment. Future research should extend the follow-up period beyond the current 12-month timeframe to deepen our understanding of long COVID recovery patterns and explore the effectiveness of specific rehabilitation interventions in improving long-term outcomes in COVID-19 survivors.

## Data Availability

The raw data supporting the conclusions of this article will be made available by the authors, without undue reservation.
